# Evaluating the Impact of Participating in KNIGHTS (Keeping Neighbors In Good Health Through Service) Clinic on Clinical Performance and Skills Development

**DOI:** 10.7759/cureus.112943

**Published:** 2026-07-19

**Authors:** Nathan M Jacob, Brandon Molligoda, Samantha Gibson, Adithi A Tirumalai, Scott Wiltz

**Affiliations:** 1 Family Medicine, University of Central Florida College of Medicine, Orlando, USA

**Keywords:** clinical competence, clinical skills assessment, experiential learning, medical education, objective structured clinical examination, osce performance, standardized patient, student-run free clinic, undergraduate medical training, underserved populations

## Abstract

Introduction

Student-run free clinics provide early clinical exposure for preclinical medical students and are associated with improved self-reported confidence and preparedness. However, there is limited objective evidence evaluating whether these experiences translate into measurable differences in standardized assessments of clinical competence. Objective Structured Clinical Examinations (OSCEs) are commonly used to assess clinical skills, though their ability to capture competencies developed in real-world clinical settings remains uncertain. This study examined the association between participation in the University of Central Florida’s KNIGHTS (Keeping Neighbors In Good Health Through Service) free clinic and OSCE performance.

Methods

Deidentified OSCE scores (0-100) were obtained for second-year medical students (MS2s) and third-year medical students (MS3s) from 2020 to 2024 and stratified by KNIGHTS volunteer status and by patient-facing roles as advanced student providers (ASP). Independent t-tests and repeated measures ANOVA were used (p < 0.05). Analyses were conducted using SPSS Statistics v. 31 (IBM Corporation, Armonk, NY, US).

Results

A total of 354 students were included in the analysis. No statistically significant differences in OSCE performance were observed between KNIGHTS volunteers (n = 113) and non-volunteers (n = 241) at either time point (MS2: 83.6 ± 10.1 vs. 83.2 ± 10.9, p = 0.75; MS3: 77.8 ± 3.8 vs. 77.3 ± 3.9, p = 0.27). Similarly, no significant differences were found between ASP (n = 40) and non-ASP (n = 73) volunteers (all p > 0.05).

Conclusion

Participation in the KNIGHTS student-run free clinic was not associated with higher MS2 or MS3 OSCE scores in this cohort. These findings should be interpreted as associations rather than causal effects. One possible explanation is that some competencies developed through real-world patient care are not fully represented in standardized OSCE outcomes. However, such competencies were not independently measured in this study.

## Introduction

Student-run free clinics (SRFCs) are outpatient clinics that are managed by medical students under faculty supervision. The scope of practice varies by clinic, but most provide free or low-cost healthcare to underserved populations in the community. These clinics offer the opportunity to not only provide care to underprivileged patients but also participate in meaningful clinical encounters even prior to starting clinical rotations. Upperclassmen also play an important role in enhancing the educational experience of preclinical volunteers at SRFCs by providing guidance, teaching, and informal evaluation [1]. Previous research has suggested that medical students report improvement in clinical competencies after volunteering with SRFCs and that they acquired skills unlikely to be gained elsewhere in the preclinical medical school curriculum [2-4]. While student-run free clinics are widely described as beneficial learning environments, concerns have also been raised regarding variability in supervision, potential risks to patients, and the ethical implications of using underserved populations as training settings. Additionally, most existing studies rely on self-reported outcomes, leaving uncertainty as to whether these experiences translate into measurable clinical competence.

The Keeping Neighbors in Good Health Through Service (KNIGHTS) Clinic is an SRFC that provides care to underserved patients in the Central Florida area. Managed by a student board from the University of Central Florida College of Medicine (UCF COM), the clinic offers an opportunity for students to gain practical experience with real patient encounters. Notably, the KNIGHTS Clinic student board consists of second-year medical students (MS2s), thus allowing students to participate in patient care prior to beginning their clinical curriculum of core and elective clerkships. This hands-on exposure complements the standardized patient (SP) interactions typical of general preclinical medical school curricula by preparing them for a level of complexity rarely found in SP encounters. Upperclassmen, including third- and fourth-year medical students (MS3 and MS4, respectively), can also volunteer with KNIGHTS Clinic but primarily serve to aid MS2 students in their clinical encounters and provide informal subjective evaluations of their performance during these encounters.

Advanced Student Providers (ASPs) are medical student volunteers within the KNIGHTS Clinic student board who are formally assigned to a direct patient care role. Although all KNIGHTS Clinic student board members receive training to function as ASPs, only a subset of students is assigned to serve routinely as the primary student providers during clinic sessions. These students, therefore, have more consistent direct patient-care responsibilities. Other board members primarily fulfill administrative, coordination, or support roles, although they may occasionally assist with patient care as needed. Our prior studies of MS2 volunteers indicated that ASPs feel better prepared for clinical simulations and real-world practice due to their consistent exposure to patient encounters [5]. They also report subjective confidence in their preparedness for their careers in medicine after volunteering with KNIGHTS Clinic, reflecting a similar trend observed with participation in other free clinics [6,7]. Additionally, we have observed an improvement in MS2 proficiency with essential clinical skills based on upperclassmen ratings of their patient encounters; however, these results are based on subjective evaluations. There are limited studies available that have objectively examined the benefits of participation with SRFCs on clinical skills development. Despite these reported benefits, there is limited objective evidence evaluating whether participation in student-run free clinics translates into measurable improvements in standardized assessments of clinical competence. Furthermore, it remains unclear whether commonly used assessment tools, such as Objective Structured Clinical Examinations (OSCEs), can capture the types of skills developed through real-world clinical experiences. These competencies may include communication across language and cultural barriers, adaptability in resource-limited settings, and management of complex social determinants of health skills that may not be fully captured in standardized clinical examinations.

The OSCE is an objective assessment commonly utilized at medical schools to assess medical students' clinical competencies in a controlled setting. Although varied across institutions, OSCEs generally involve a multi-station setup in which students encounter standardized patients (SPs) in timed, simulated clinical encounters. Students are given a clinically relevant task at each station such as taking a history, performing relevant physical examinations, or writing a pertinent post-encounter note. During these interactions, students are assessed based on their efficacy in completing these tasks as well as their communication skills with SPs.

At UCF COM, OSCE assessments are used at the end of each academic year for MS1-MS3 students, with the difficulty and complexity of SP encounters adjusted to the level of clinical knowledge expected of students at each academic level. To mimic real-world clinical settings, each OSCE patient room is set up as a standard clinic room with medical equipment available to use at the student’s discretion such as ophthalmoscopes, otoscopes, and reflex hammers. Students are individually assessed at each station by faculty using a standardized rubric that examines their proficiency in several core competencies, including communication skills, professionalism, and clinical reasoning. Students are then given a composite score (ranging from 0 to 100 points) that represents their overall performance across all stations. Prior to taking their OSCE, MS2 students have completed their preclinical coursework, and MS3 students have completed their core clerkships, which include pediatrics, obstetrics and gynecology, internal and family medicine, surgery, neurology, and psychiatry.

The primary objective of this study was to evaluate the association between participation in the KNIGHTS student-run free clinic during the MS2 year and subsequent performance on standardized MS2 and MS3 OSCEs among UCF College of Medicine students. OSCE scores were used as standardized measures of selected competencies, including history taking, physical examination, communication, professionalism, and clinical reasoning, rather than as comprehensive measures of real-world clinical competence. A secondary exploratory aim was to examine whether associations differed according to the degree of patient-facing responsibility, as represented by Advanced Student Provider status.

## Materials and methods

This study utilized a retrospective cohort design. This study was reviewed by the University of Central Florida Institutional Review Board and determined to be exempt from human subjects research regulations (IRB ID: STUDY00006210). The requirement for informed consent was waived due to the use of de-identified data.

We obtained MS2 and MS3 end-of-year OSCE scores from 2020 to 2024 to assess whether there was an immediate association between volunteering and clinical performance (MS2 scores) as well as a potential longitudinal association (MS3 scores). OSCE stations assessed domains including history taking, physical examination, communication skills, professionalism, and clinical reasoning using standardized scoring rubrics. While OSCE formats were consistent across years in structure and assessed domains, minor variations in case content and examiners may have occurred.

The UCF COM Office of Assessment de-identified each score and assigned each student a unique identifier linking their MS2 and MS3 OSCE scores. Students were stratified based on participation in KNIGHTS Clinic during their MS2 year (“KNIGHTS” vs. “non-KNIGHTS”), with KNIGHTS volunteers further stratified by role. ASP status reflected a formally assigned clinic role rather than a threshold based on hours or number of patient encounters. Students assigned to the ASP role routinely served as primary student providers with consistent direct patient-care responsibilities, whereas non-ASP volunteers primarily fulfilled administrative, coordination, or support roles and could occasionally participate in patient-facing activities.

For the present analysis, KNIGHTS Clinic participation was categorized according to volunteer status and assigned clinic role. Although participation varied in frequency and intensity, cumulative measures such as the number of clinic sessions, patient encounters, or service hours were not incorporated into the current analysis.

Continuous variables are reported as mean ± standard deviation. The primary analyses compared MS2 and MS3 OSCE scores between students who participated in KNIGHTS Clinic during the MS2 year and students who did not participate. Secondary analyses compared ASP and non-ASP volunteers and, exploratorily, the three groups of non-KNIGHTS students, non-ASP volunteers, and ASP volunteers. Two-group comparisons (KNIGHTS vs. non-KNIGHTS; ASP vs. non-ASP) were performed using independent samples t-tests, the exploratory three-group comparison was performed using one-way ANOVA, and within-student change across assessment years was evaluated using repeated-measures ANOVA. Analyses were conducted using SPSS Statistics v31 (IBM Corp., Armonk, NY, US).

Because MS2 and MS3 OSCE scores were available for the same students, within-student differences across the two assessment time points were evaluated using a repeated-measures ANOVA with assessment year as the within-subject factor and volunteer group as the between-subject factor. Because the MS2 and MS3 OSCEs differed in content and level-specific expectations, this analysis was interpreted as a comparison of scores across two related but non-equivalent assessments rather than as a direct measure of longitudinal change in clinical competence. Only students with available paired MS2 and MS3 OSCE scores were included in the longitudinal analysis.

Statistical significance was defined as a two-sided p-value < 0.05. Effect sizes and 95% confidence intervals were reported where appropriate. Secondary analyses were considered exploratory. Because this was a retrospective analysis of all eligible students with available paired OSCE data during the study period, no a priori sample size calculation was performed. The available sample size was determined by the number of eligible students meeting the study inclusion criteria. The present analysis did not include adjustment for baseline academic performance, prior healthcare experience, motivation, or participation in other clinical or OSCE preparation activities.

## Results

**Figure 1:**
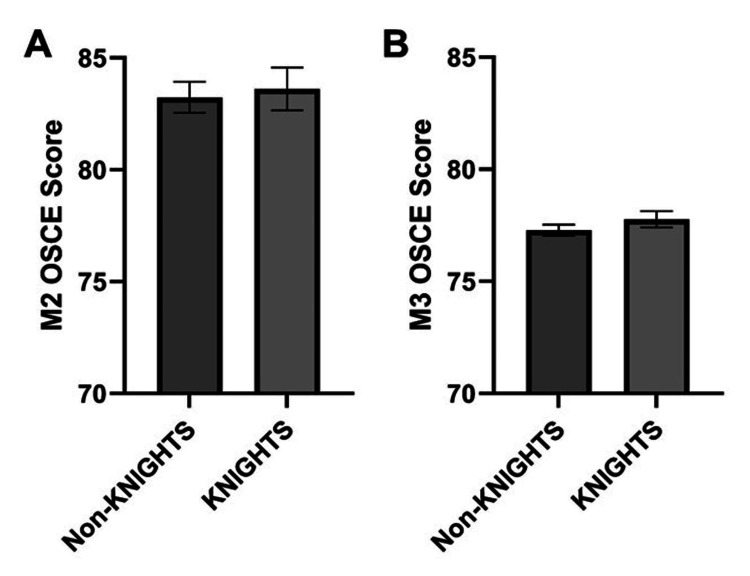
Comparison of mean OSCE scores by KNIGHTS Clinic participation and volunteer role Comparison of mean OSCE scores from (A) MS2s and (B) MS3 based on participation in KNIGHTS Clinic. No statistically significant differences were found for either set of scores (p > 0.05). OSCE = Objective Structured Clinical Examination; MS2 = second-year medical student; MS3 = third-year medical students; ASP = Advanced Student Provider; KNIGHTS = Keeping Neighbors in Good Health Through Service

A total of 354 paired MS2 and MS3 OSCE scores were analyzed (Figure 1). There were no statistically significant differences in OSCE performance between KNIGHTS Clinic volunteers (n = 113) and non-volunteers (n = 241) at either time point. Mean MS2 OSCE scores were 83.6 ± 10.1 among KNIGHTS volunteers and 83.2 ± 10.9 among non-volunteers, corresponding to a mean difference of 0.4 points (95% CI: −2.0 to 2.8; effect size: 0.04; p = 0.75). Mean MS3 scores were 77.8 ± 3.8 and 77.3 ± 3.9, respectively, corresponding to a mean difference of 0.5 points (95% CI: −0.4 to 1.3; effect size: 0.13; p = 0.27).

**Figure 2:**
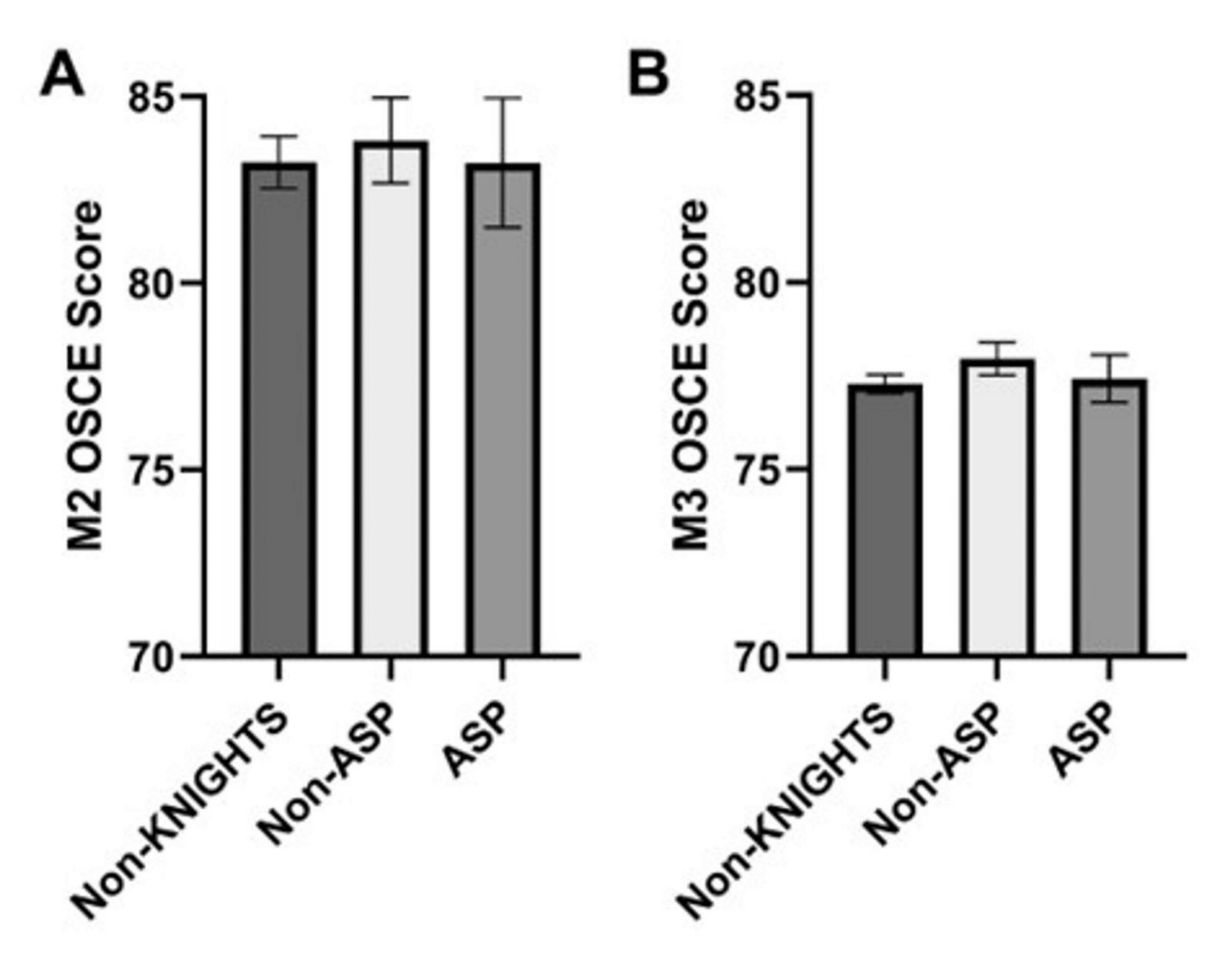
Comparison of mean OSCE scores by KNIGHTS Clinic participation and volunteer role Comparison of mean OSCE scores from (A) second-year medical students (MS2) and (B) third-year medical students (MS3) based on participation and role in KNIGHTS Clinic. No statistically significant differences were found (all p > 0.05). OSCE = Objective Structured Clinical Examination; ASP = Advanced Student Provider; KNIGHTS = Keeping Neighbors in Good Health Through Service

Among KNIGHTS Clinic volunteers, no statistically significant differences in OSCE performance were observed between ASP (n = 40) and non-ASP (n = 73) students (Figure 2). Mean MS2 scores were 83.2 ± 10.9 among ASPs and 83.8 ± 9.7 among non-ASPs, corresponding to a mean difference of -0.6 points (95% CI: -4.6 to 3.4; effect size: -0.06; p = 0.77). Mean MS3 scores were 77.4 ± 4.0 and 78.0 ± 3.7, respectively, corresponding to a mean difference of -0.5 points (95% CI: -2.0 to 1.0; effect size: -0.14; p = 0.48). Comparisons between ASPs, non-ASPs, and non-volunteers were not statistically significant (all p > 0.05).

**Figure 3:**
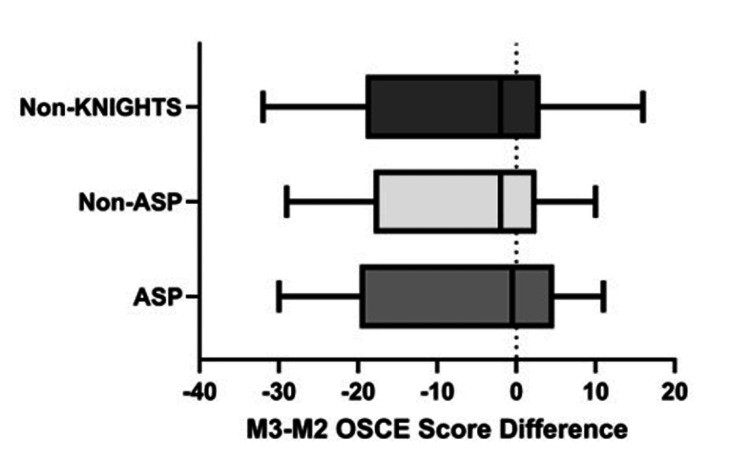
Difference in OSCE scores between MS3 and MS2 students by volunteer history Difference between MS3 and second-year medical student MS2 OSCE scores for each subject based on volunteer history. As discussed in the text, the MS2 and MS3 OSCEs were not vertically equated. A dashed line is shown at the OSCE score difference = 0. OSCE = Objective Structured Clinical Examination; MS2 = second-year medical student; MS3 = third-year medical students; ASP = Advanced Student Provider; KNIGHTS = Keeping Neighbors in Good Health Through Service

Across all groups, MS3 OSCE scores were lower than MS2 scores (Figure 3). The difference between MS2 and MS3 scores was statistically significant within each cohort (all p < 0.01).

We additionally examined whether the pattern of OSCE scores across the MS2 and MS3 assessments differed according to KNIGHTS Clinic participation and assigned volunteer role. To investigate this, we performed a repeated-measures ANOVA to identify factors contributing to score changes between the MS2 and MS3 assessments (Table 1), which demonstrated a significant effect of assessment year on OSCE performance (F(1, 351) = 50.8, p < 0.01, η² = 0.07). There was no significant effect of volunteer status (F(2, 351) = 0.4, p = 0.67, η² = 0.001) and no significant interaction between assessment year and volunteer status (F(2, 351) = 0.004, p > 0.99, η² ≈ 0.00001).

**Table 1:** Repeated measures ANOVA examining the effects of volunteer history and training year on OSCE performance * Statistically significant DFn = degrees of freedom for numerator; DFd = degrees of freedom for denominator; η² = partial eta squared, proportion of variance explained. The model included assessment year as the within-subject factor and volunteer group as the between-subject factor. Effect sizes are reported as partial eta squared (partial η²). ANOVA: Analysis of Variance; OSCE = Objective Structured Clinical Examination

Effect	DFn	DFd	F	p-value	η^2^
Intercept	1	351	47874.99	< 0.01*	0.98
Volunteer History	2	351	0.3942	0.67	0.001
Year	1	351	50.82	< 0.01*	0.07
Volunteer History: Year Interaction	2	351	0.0036	> 0.99	0.00001

## Discussion

This study evaluated the association between participation in a student-run free clinic and OSCE performance among medical students and found no statistically significant differences between volunteers and non-volunteers. Because clinic participation was self-selected and the analyses were unadjusted for several potential confounders, the results should not be interpreted as evidence of a causal effect or lack of effect of clinic participation on clinical competence. One possible interpretation is that some competencies developed through experiential learning environments may not be fully represented in the standardized OSCE outcomes examined in this study. However, because such competencies were not independently measured, the present study cannot directly determine whether a mismatch exists between clinic-based learning experiences and the assessment tool.

Our findings are consistent with prior work by Nakamura et al., who similarly found no significant association between student-run clinic participation and OSCE performance despite differences in self-reported confidence [8]. In contrast, Seifert et al. demonstrated improved outcomes when structured training interventions were incorporated into student-run clinic participation [9]. Together, these findings suggest that while experiential learning environments provide valuable exposure, their association with standardized assessment outcomes may depend on the degree of alignment with curricular objectives and evaluation methods.

Participation in student-run free clinics may foster competencies that are not routinely captured in OSCE formats, including adaptability in resource-limited settings, communication across language and cultural barriers, and the ability to navigate complex social determinants of health. Prior studies have demonstrated improvements in self-confidence, attitudes toward underserved populations, and perceived quality of care among student volunteers [10-13]. These skills are essential to real-world clinical practice but may not be reflected in structured, time-limited simulated encounters. Accordingly, the absence of measurable differences in OSCE performance should not be interpreted as evidence that clinic participation lacks educational value. It instead leaves open the possibility that educational effects may occur in domains not captured by the OSCE outcomes examined here. However, this possibility was not directly tested in the present study.

OSCEs are widely used in health professions education because they provide a standardized approach to assessing predefined clinical tasks and domains such as communication, history taking, physical examination, professionalism, and clinical reasoning. When appropriately designed, OSCEs can provide useful and structured evidence of learner performance. At the same time, OSCE scores may be influenced by station sampling, case content, examiner behavior, scoring procedures, and the number of stations included [14-16]. Inter-rater variability may also introduce measurement error, particularly when multiple faculty examiners are involved. Inter-rater reliability estimates were not available for the examinations analyzed in this study and, therefore, could not be incorporated into the analysis. Additionally, both students and faculty have expressed concerns regarding whether OSCEs accurately reflect real-world clinical competence [17,18].

The absence of an association between KNIGHTS participation and OSCE scores may therefore have several possible explanations. Clinic participation may not produce meaningful differences in the competencies emphasized by the institutional OSCE. Any differences may have been too small to detect precisely, heterogeneity in participation intensity may have diluted associations, residual confounding may have influenced the comparisons, or some competencies developed through student-run clinic participation may not have been emphasized in the OSCE domains assessed. The present study cannot distinguish among these explanations.

It is important to acknowledge ethical considerations related to student-run free clinics. Student-run free clinics serve populations that may experience barriers to healthcare and must therefore prioritize patient welfare, informed consent, appropriate supervision, continuity of care, and community benefit. Educational value alone is insufficient to establish the success of these programs. Although the present study focused on learner outcomes, future evaluations should also incorporate patient-centered measures, including quality of care, patient experience, continuity, access to services, and community impact. Combining learner-centered and patient-centered outcomes may provide a more complete assessment of the value and appropriateness of student-run clinic programs.

We observed lower numerical scores on the MS3 OSCE than on the MS2 OSCE across all groups. However, the two assessments were designed for different stages of training and differed in content, complexity, expectations, and potentially examiner context. They should therefore not be treated as vertically equivalent measures of a single underlying scale, and the observed score difference should not be interpreted as a decline in clinical competence. Accordingly, the repeated-measures analysis is best interpreted as examining whether score patterns across two related but non-equivalent assessments differed by volunteer group, rather than as a direct measure of longitudinal clinical growth or decline.

Because standardized assessments like the OSCE may not fully capture competencies developed through real-world patient care, future studies may benefit from exploring alternative or modified assessment strategies that better align with experiential learning environments. One potential direction would be the development and piloting of OSCE stations specifically designed to assess cultural competency, health equity awareness, and communication across language or socioeconomic barriers. Such assessments may more closely mirror not only the types of encounters in student-run clinics, but also common, real-world encounters more globally. This could provide both more sensitive measures of the educational value of these programs and opportunities to increase whole-person care in medical school curricula.

Limitations

This study has several limitations. First, it was conducted at a single institution and evaluated participation in a single student-run free clinic, which may limit generalizability to programs with different clinical structures, supervision models, or student responsibilities.

Second, participation in KNIGHTS Clinic was voluntary rather than randomized, creating the potential for selection bias and residual confounding. Students who elected to participate, and particularly those assigned to patient-facing roles, may have differed from non-volunteers in motivation, prior healthcare experience, baseline clinical confidence, academic performance, specialty interests, or participation in other clinical and OSCE preparation activities. These variables were not included in the present analysis, and the findings should therefore be interpreted as unadjusted associations rather than causal effects of clinic participation.

Third, clinic participation was categorized according to volunteer status and assigned role rather than cumulative exposure. Students within the same category may have differed in the number of clinic sessions attended, patient encounters completed, and total service hours. This exposure heterogeneity may have attenuated associations between clinic participation and OSCE performance. Future studies should incorporate quantitative measures of participation intensity and examine potential dose-response relationships.

Fourth, ASP status reflected an assigned clinic role rather than a standardized cumulative measure of direct patient contact. Although all board members received ASP training, students assigned to the ASP role routinely provided direct patient care, whereas other volunteers primarily fulfilled administrative, coordination, or support responsibilities and could occasionally participate in patient-facing activities. Some overlap in clinical exposure between groups may therefore have occurred.

Fifth, although the overall cohort included 354 students, some subgroup analyses, particularly those involving ASPs, included substantially fewer participants and may have had limited precision for detecting small between-group differences. The confidence intervals around the estimated effects should therefore be considered when interpreting the null findings.

Sixth, OSCE scores represent performance on structured standardized assessments and should not be interpreted as comprehensive measures of real-world clinical competence. Examiner variation and the unavailability of inter-rater reliability data, noted above, are additional sources of measurement uncertainty.

Finally, the MS2 and MS3 OSCEs were designed for different stages of training and differed in case content, complexity, and expected performance. They were not vertically equated. Therefore, numerical score differences across the two assessments should not be interpreted as direct measures of longitudinal improvement or decline in clinical competence.

Future studies should incorporate larger multi-institutional cohorts, quantitative measures of clinic exposure, baseline academic and experiential characteristics, adjusted analyses, and outcomes specifically designed to assess competencies emphasized in real-world service-learning environments.

## Conclusions

Participation in the KNIGHTS student-run free clinic was not associated with higher MS2 or MS3 OSCE scores in this retrospective cohort. Similarly, assignment to the patient-facing ASP role was not associated with higher standardized assessment scores. These findings represent unadjusted associations and should not be interpreted as evidence for or against a causal effect of clinic participation on broader clinical competence. Several explanations for the null findings remain possible, including heterogeneity in participation intensity, residual confounding, limited precision for detecting small differences, and differences between competencies emphasized in real-world clinical environments and those assessed by institutional OSCEs. Future studies should incorporate quantitative measures of clinical exposure, baseline learner characteristics, and both learner-centered and patient-centered outcomes.

## References

[1] Schutte T, Tichelaar J, Dekker RS, van Agtmael MA, de Vries TP, Richir MC (2015). Learning in student-run clinics: a systematic review. Med Educ.

[2] Smith SD, Johnson ML, Rodriguez N, Moutier C, Beck E (2012). Medical student perceptions of the educational value of a student-run free clinic. Fam Med.

[3] Adel FW, Berggren RE, Esterl RM Jr, Ratelle JT (2021). Student-run free clinic volunteers: who they are and what we can learn from them. BMC Med Educ.

[4] Huang K, Maleki M, Regehr G, McEwen H (2021). Examining the educational value of student-run clinics for health care students. Acad Med.

[5] Kalistratova VS, Nisanova A, Shi LZ (2024). Student-run free clinics may enhance medical students' self-confidence in their clinical skills and preparedness for clerkships. Med Educ Online.

[6] Choudhury N, Khanwalkar A, Kraninger J, Vohra A, Jones K, Reddy S (2014). Peer mentorship in student-run free clinics: the impact on preclinical education. Fam Med.

[7] Ng E, Hu T, McNaughton N, Martimianakis MA (2021). Transformative learning in an interprofessional student-run clinic: a qualitative study. J Interprof Care.

[8] Nakamura M, Altshuler D, Chadwell M, Binienda J (2014). Clinical skills development in student-run free clinic volunteers: a multi-trait, multi-measure study. BMC Med Educ.

[9] Seifert LB, Schaack D, Jennewein L, Steffen B, Schulze J, Gerlach F, Sader R (2016). Peer-assisted learning in a student-run free clinic project increases clinical competence. Med Teach.

[10] Smith SD, Yoon R, Johnson ML, Natarajan L, Beck E (2014). The effect of involvement in a student-run free clinic project on attitudes toward the underserved and interest in primary care. J Health Care Poor Underserved.

[11] Lessans S, Bogdanovski K, Porter KR, Ballantyne K, Pasarica M (2021 (2021). Service on student-run free clinic executive board improves leadership skills of medical students in the underserved medical system. Leadersh Health Serv (Bradf Engl).

[12] Ballantyne K, Porter KR, Bogdanovski K, Lessans S, Pasarica M (2021). Cultural sensitivity and learning about healthcare equity for the underserved: experiential learning in a student-run free clinic. Med Sci Educ.

[13] Brown A, Ismail R, Gookin G, Hernandez C, Logan G, Pasarica M (2017). The effect of medical student volunteering in a student-run clinic on specialty choice for residency. Cureus.

[14] Brannick MT, Erol-Korkmaz HT, Prewett M (2011). A systematic review of the reliability of objective structured clinical examination scores. Med Educ.

[15] Khan KZ, Gaunt K, Ramachandran S, Pushkar P (2013). The Objective Structured Clinical Examination (OSCE): AMEE Guide No. 81. Part II: organisation & administration. Med Teach.

[16] Alkhateeb N, Salih AM, Shabila N, Al-Dabbagh A (2022). Objective structured clinical examination: challenges and opportunities from students' perspective. PLoS One.

[17] Ahmed N, Aziz S, Jouhar R (2024). Analysis of satisfaction levels and perceptions of clinical competency: a mixed method study on objective structured clinical examinations in undergraduate dental students. BMC Med Educ.

[18] Yeates P, Maluf A, Kinston R (2025). A realist evaluation of how, why and when objective structured clinical exams (OSCEs) are experienced as an authentic assessment of clinical preparedness. Med Teach.

